# CASE REPORT Removal of Exposed Titanium Reconstruction Plate After Mandibular Reconstruction With a Free Fibula Osteocutaneous Flap With Large Surgical Pin Cutters: A Case Report and Literature Review

**Published:** 2012-08-31

**Authors:** Travis G. Boyd, Katherine M. Huber, Daniel E. Verbist, Jeffery M. Bumpous, Bradon J. Wilhelmi

**Affiliations:** ^a^School of Medicine; ^b^Department of Otolaryngology; ^c^Department of Plastic and Reconstructive Surgery, University of Louisville, Ky

## Abstract

Locking reconstruction plates have led to significant improvement in osteosynthesis and graft anchorage in mandibular reconstruction following the free fibula osteocutaneous flap. Plate extrusion is the most common complication associated with mandibular reconstruction, occurring in approximately 20% to 48% of cases; often necessitating plate removal once the bone flap has united to the mandible. Radiation therapy is a known risk factor to the development of such a complication and it presents further challenges to the successful removal of the reconstruction plate. Several reports have been published regarding plate removal in the setting of orthopedics that describe the management of jammed or stripped locking screws, but few in the setting of mandibular reconstruction. In this case, we report the successful removal of an exposed titanium mandibular reconstruction plate from a 41-year-old woman 12 months after her initial reconstruction with a free fibula osteocutaneous flap and radiation therapy. The approach was selected because the chin and neck skin could not be expected to be raised for full plate exposure secondary to radiation-induced skin changes (thinning and friability). We also discuss the use of previously employed methods of plate removal in various settings as well as their inherent strengths and weaknesses.

## INTRODUCTION

Reconstruction of the mandible following malignancy has advanced significantly over the years with the aim to restore function and aesthetic outcome. The free fibular osteocutaneous flap is the preferred method of mandibular reconstruction as it provides ample bone for the reconstruction and able to handle multiple osteotomies in addition to providing soft-tissue coverage.^1^ At the same time, osteosynthesis has been enhanced through the use of titanium reconstruction plates and locking screws, as they provide excellent anchorage and achieve rigid fixation to the bone graft without compressing it, thereby reducing the risk of ischemic bone graft loss.^2^^,^^3^

The advances in micro-vascular flaps and plating systems are balanced with the treatment of the primary malignancy; this, of course, takes precedence over the reconstruction. Radiation therapy has been demonstrated to reduce the vascular supply to tissue by inducing proliferation within the subendothelial layers within vessels. The progressive occlusion of the vessels leads to tissue hypoxia and eventually ischemia that lead to cutaneous and subcutaneous atrophy and fibrosis. Clinically, this manifests itself as thin, friable skin that heals poorly.^2^^,^^4^ This is clearly demonstrated in the setting of mandibular reconstruction by the all too common complication of plate extrusion, which occurs in approximately 20–48% of cases and is associated with radiation therapy.^5^^,^^6^ This presents a significant problem for the reconstructive surgeon even after the viability of the free flap has been assured, as there are potentially many late complications that can arise and lead to poor functional and aesthetic results.

There are very few reports to date on the proper management of exposed plating systems. Several technical issues with plating systems have been addressed in the literature, mostly in the setting of trauma and orthopedics.^3^^,^^7^ While the complications with plating systems used in orthopedics typically are not due to wound healing in irradiated tissue, the complications incurred with these plating systems can undoubtedly occur in mandibular reconstruction as well. Notably, jammed screws, stripped screw heads and difficultly removing the plate itself are more common than they are reported. These complications lead to significant delays in operating time and damage to surround tissues upon their removal if not carefully executed.^6^ Therefore, it is necessary to explore all possible options than can be employed for effectively removing plating systems when the first line mechanisms fail or are not available.

This case serves to describe a straight-forward approach to removing a portion of an exposed titanium reconstruction plate when other methods have either failed or not acceptable. The approach used in the following patient was selected because of extensive post-radiation changes to the skin of her chin and neck. The high likelihood of skin necrosis and poor wound healing made removing the plate directly without damaging the surrounding soft tissue desirable. Several previously employed methods of plate removal, in various settings as well as their inherent strengths and weaknesses will also be discussed.

## CASE REPORT

This patient is a 41 year old female that underwent a composite mandibulectomy with resection of the oral floor for squamous cell carcinoma. She was immediately reconstructed with free fibula osteocutaneous flap for her mandibular and oral floor defect. The free fibula flap was harvested from her right leg preserving 6 cm of fibula proximally and distally for knee and ankle stability. A large, 14 × 18 cm skin paddle was harvested along with the fibula flap to reconstruct the floor of her mouth, the defect of which was extensive. The entire skin paddle survived and she went on to achieve bony union of both sides of the fibula graft and the single osteotomy. Fixation of the fibula to the mandible was performed using a Stryker Locking Reconstruction Plate from the right mandibular ramus to the left angle of the mandible. The vascular microanastomosis between the Peroneal artery and vein of the fibular flap was achieved in an end-to-side manner with the External Carotid Artery and Internal Jugular Vein, respectively. Her initial postoperative course was uneventful. She eventually began eating well, speaking and breathing without difficulty and her feeding tube and tracheostomy were removed. She did however require postoperative radiotherapy to the mandible, oral floor, and neck.

Twelve months postoperatively, she developed thinning of the chin skin and exposure of the anterior portion of the mandibular plate. CT scan confirmed bony union of the free fibula to the mandible bilaterally. Her skin was very thin throughout the neck and, interestingly, the skin had epithelialized under the exposed reconstruction plate. At this time it was decided to approach her plate directly and in a manner that would safely not disrupt the bone graft and epithelialized skin below the reconstruction plate. A small extension of the skin opening was performed in the lateral direction on both sides. The exposed screws were removed transcutaneously. The titanium reconstruction plate was then removed with a single cut on both sides, immediately proximal to the point of initial exposure using a Large Surgical Pin Cutter. The plate was then removed and the skin was closed in simple fashion with 5-0 nylon sutures that were removed 2 weeks later. The wound went on to heal well.

## DISCUSSION

Radiation therapy damages small vessels by reducing smooth muscle density and progressively thickening the sub-endothelial components of vessel wall, leading to progressive occlusion and fibrosis of the vessels.^4^^,^^8^ On top of threatening the perfusion of the flap, radiation induces changes of skin and subcutaneous tissues that lead skin atrophy and firability.^4^ These changes arise from decreased mitotic activity of the epidermis that leads to the loss of keratinized layers as well as depletion of stem cells within the stratum basalis.^4^ This concept is demonstrated in the above case report plate extrusion is so common in the setting mandibular reconstruction with reconstruction plates.

Preventing late complications that arise from wound healing in irradiated tissue is currently a highly researched field and several studies have demonstrated improved outcomes with decreased rates of ulceration by pre-treating subcutaneous tissues prior to radiation. Of these Amifostine, Sucralafate, Fibroblast Growth Factor (FGF) and Platelet Rich Plasma (PRP) are all currently being investigated as possible methods to prevent the aforementioned complications.^4^^,^^9^^-^12. Unfortunately, there have not been any conclusive studies at this time that clearly demonstrate the usefulness of any one method over another for the prevention and management of radiation induced soft tissue changes. Therefore, at this time, selection of a free flap and ensuring reliable blood supply is the still the most efficient means of preventing complications to reconstruction in an irradiated field.

In comparison to the numerous studies that illustrate the potential complications associated with locking plates in the setting of mandibular reconstruction, a comparatively small number of studies have commented on how to effectively manage complications arising from locking plates in general. Ehlinger et al. reported on technical difficulties in hardware removal with titanium compression plates with locking screws in an orthopaedic setting. This study describes various complications such as the screw jamming, screw head destruction either through the recess being stripped or filled and issues in removing the plate itself. For instance, in the case of a jammed screw, they recommend using a tungsten drill to completely destroy the screw head in order to enlarge the plate hole, and then using a trephine drill to extract the remaining screw body. Striped locking screws can be removed with a conical left-turn extraction screwdriver or a tungsten drill and plates can be removed using lever arm maneuvers.

The carbide burr has also been found to be a reliable method for removing jammed cold-welded locking screws. This method entails burring through the screw until the plate disengages.^13^ As one might imagine this method generates a great deal of heat and debris, which can easily cause injury to surrounding soft tissue. If this method is used, it is recommended that plenty of irrigation is used and the surrounding soft tissue is protected.

Kumar et al. described removed jammed locking screws from a Less Invasive Stabilizing System (LISS) titanium plate using a high-speed disc.^14^ A radial cut from the plate edge to the screw hole was made and a 10~mm osteotome was wedged into the cut to increasing the circumference of the threaded portion of the screw hole. The locking screw was eventually removed with a conical extraction screw.^12^ This method took a great deal of time and used several high-speed discs in order to safely cut from the edge of the reconstruction plate to the screw-hole, being careful not to cut into the screw head itself. Time was also dedicated to making sure that excessive heat was not generated while cutting with the high-speed disc. As indicated by the long operating time (approximately 3.5 hours in the aforementioned study), extreme precision is required since there is a chance that the high-speed disc could slice though the plate into the underlying bone, negating the need for plate removal.

Lehman et al. developed a technique to remove cold- welded titanium locking screws. Their method entails connecting a Jacob's T-handle chuck to a Synthes star drive shaft and then using a mallet to incarcerate the screw driver into the screw head in order to provide enough torque to remove the cold-welded locking screws.^13^ This is an effective and soft-tissue friendly method of screw removal and it certainly has its place in the setting of a jammed locking screw.

Our method utilized a Large Surgical Pin Cutter to remove a titanium reconstruction while leaving the unexposed parts of the plate intact without disrupting the adjacent soft tissue. Minimal damage to the surrounding skin and soft-tissue was of the utmost importance in this case due to the poor likelihood that a large wound would heal reliably in a previously irradiated field. Bony union at the bridging point between the fibular graft and mandible was confirmed via CT scan; thereby ensuring that the plate would be able to be safely removed without sacrificing stability of the mandible. After removal, the splice points of the plates were smooth with no evidence of kinking, which can occur if too much leverage is placed on the plate or if a poor initial cut is made.

Large Surgical Pin Cutters are bulky, which limits their precision and maneuverability; however, in this case, injury to the underlying skin and bone was unacceptable and pin cutters were a safe way of removing the plate without risking damage to the underlying tissue. While high- speed discs and carbide burrs cut through titanium plates and locking screws with relative ease, the production of extreme temperatures and debris were undesirable in this case, as they likely would have damaged the underlying tissues. Large surgical pin cutters, although limited in maneuverability, are an effective alternative that produces no excess heat and, as in this case, are able to remove exposed titanium reconstruction plates in a single piece.

## Figures and Tables

**Figure 1:**
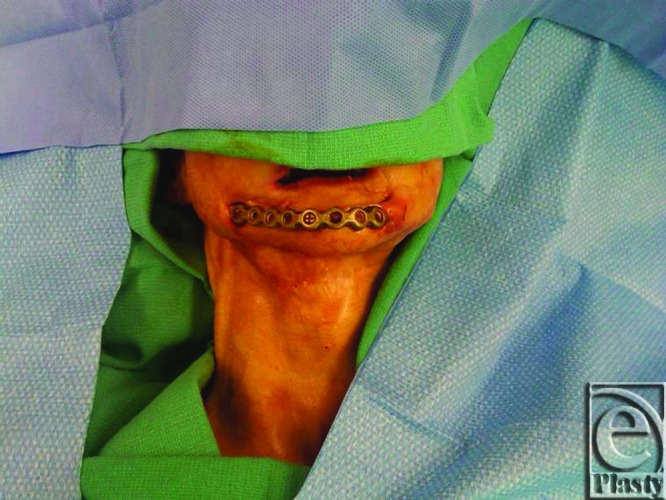
Preoperative photograph of a patient with exposed reconstruction plate, 1 year following mandibular reconstruction with a free fibular osteocutaneous flap.

**Figure 2:**
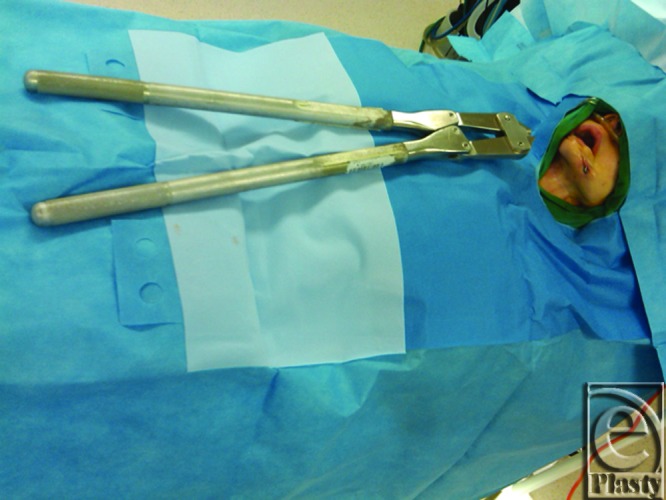
Large surgical pin cutters.

**Figure 3:**
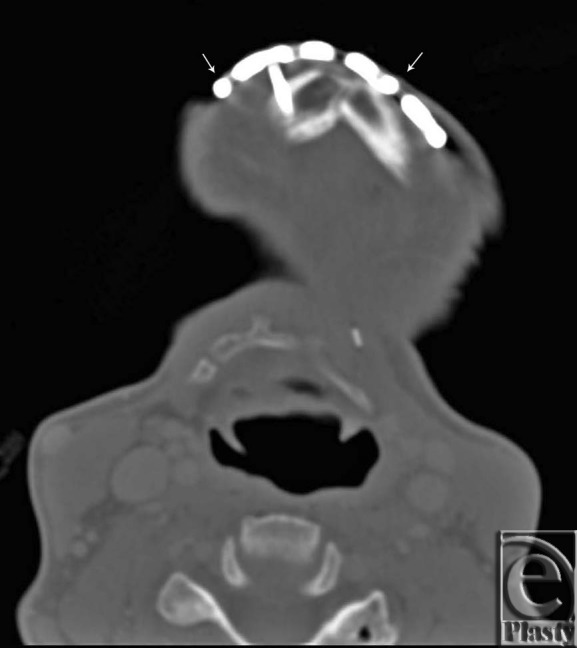
Preoperative computed tomographic scan. Arrows indicated where cuts were made with large surgical pin cutters to remove the exposed portion of the reconstruction plate.

**Figure 4:**
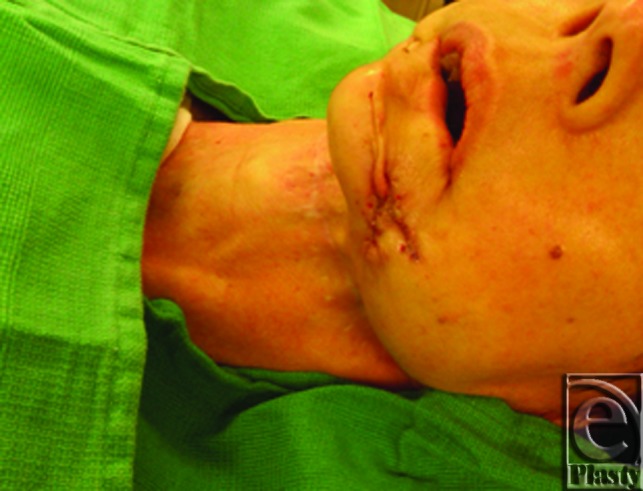
Postoperative photograph of a patient following plate removal with large surgical pin cutters.
